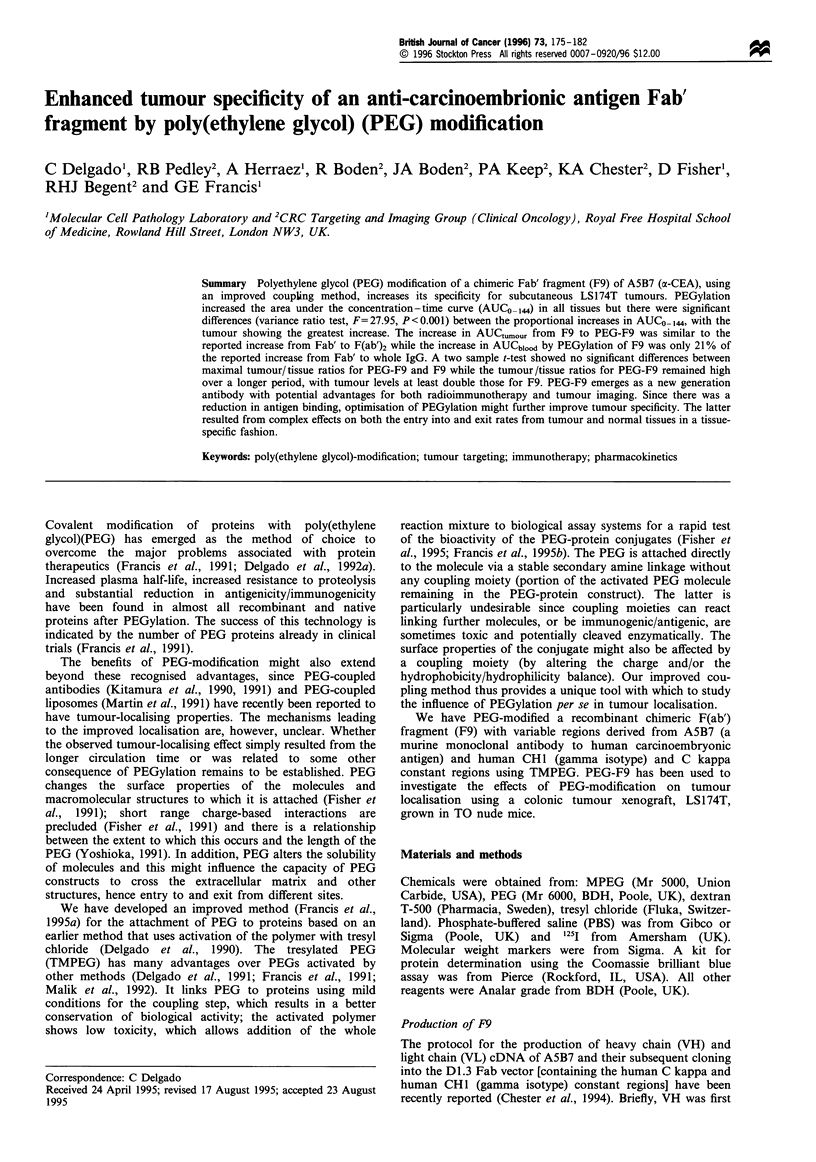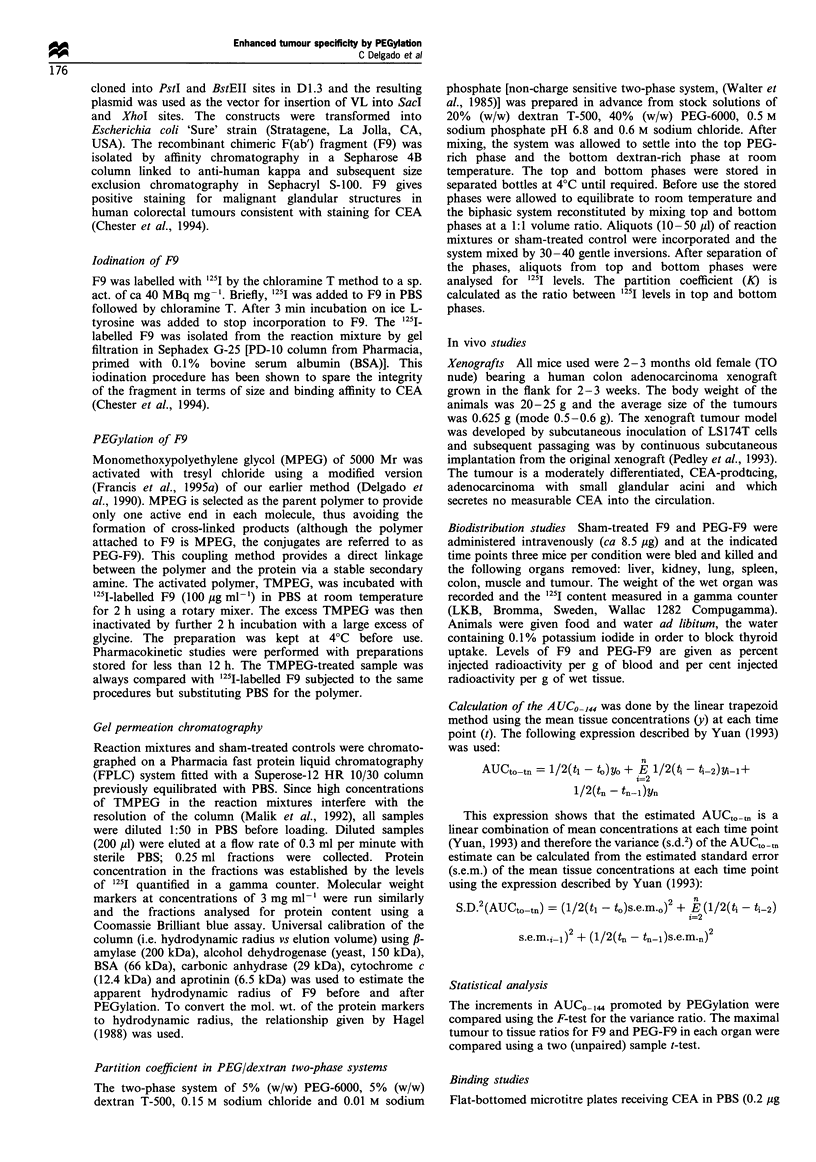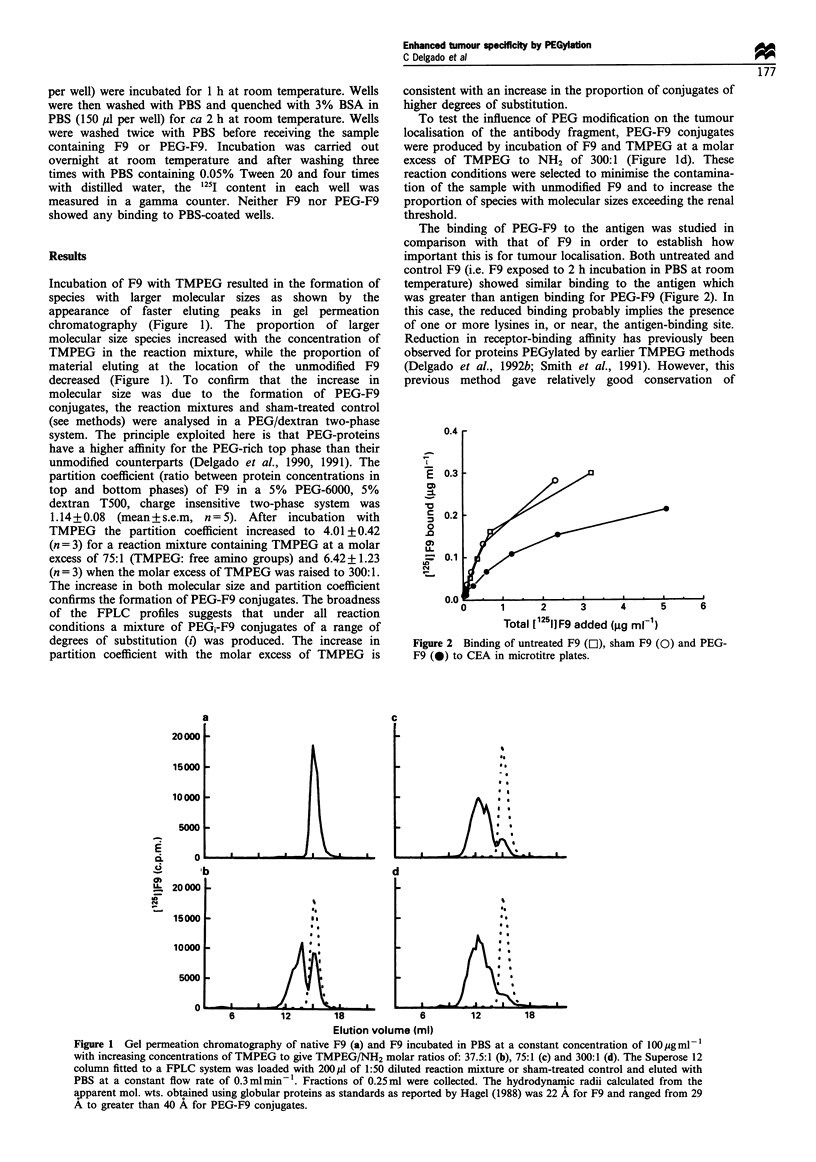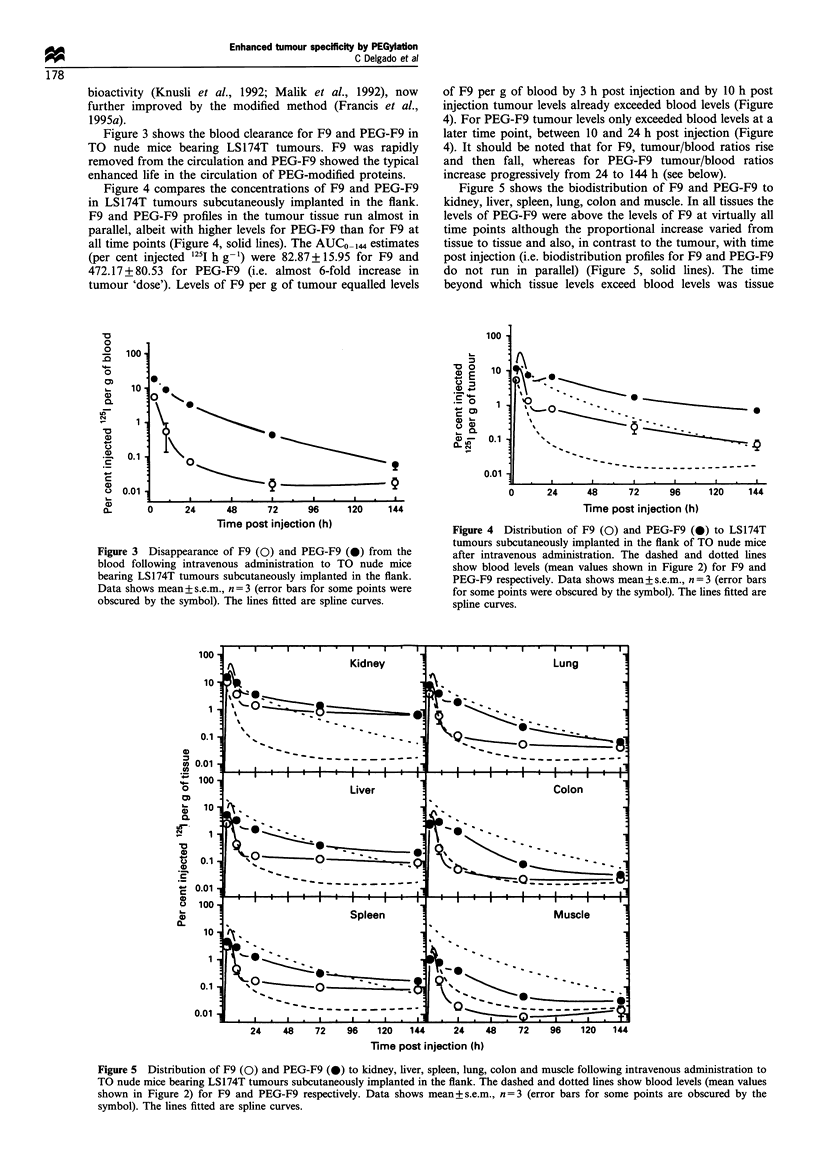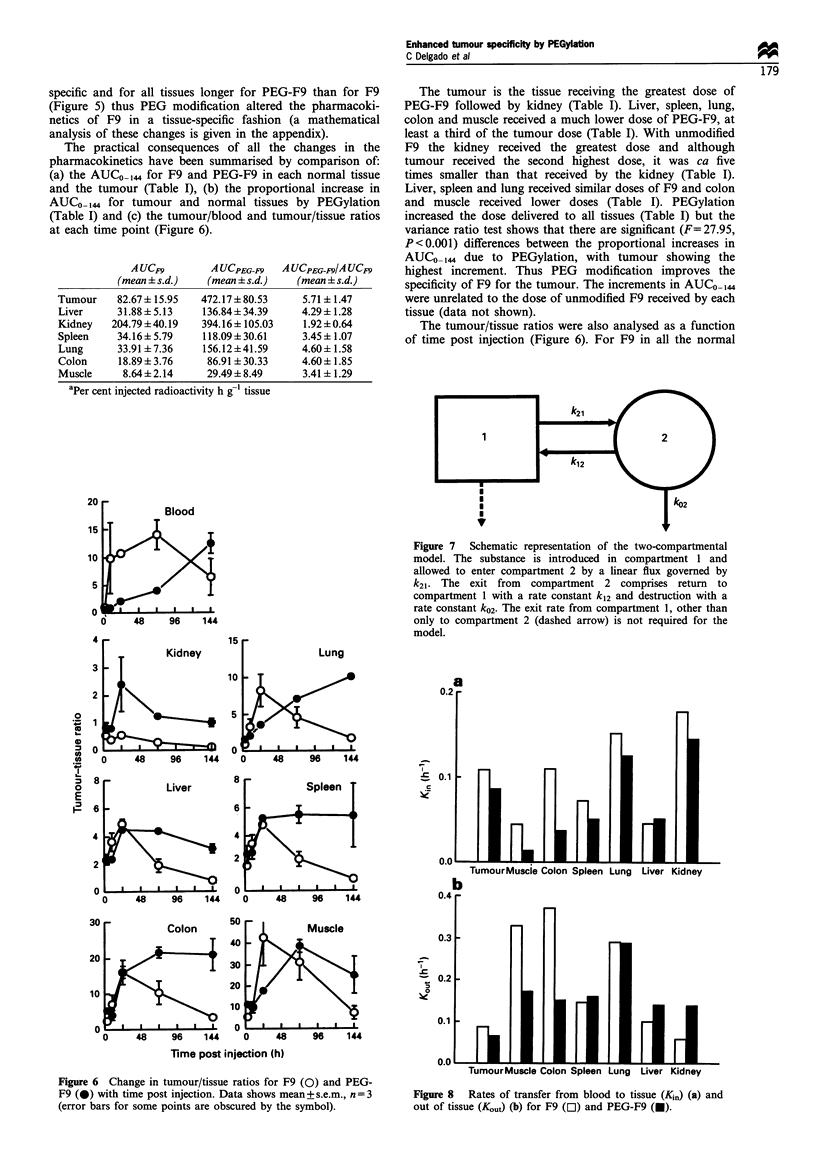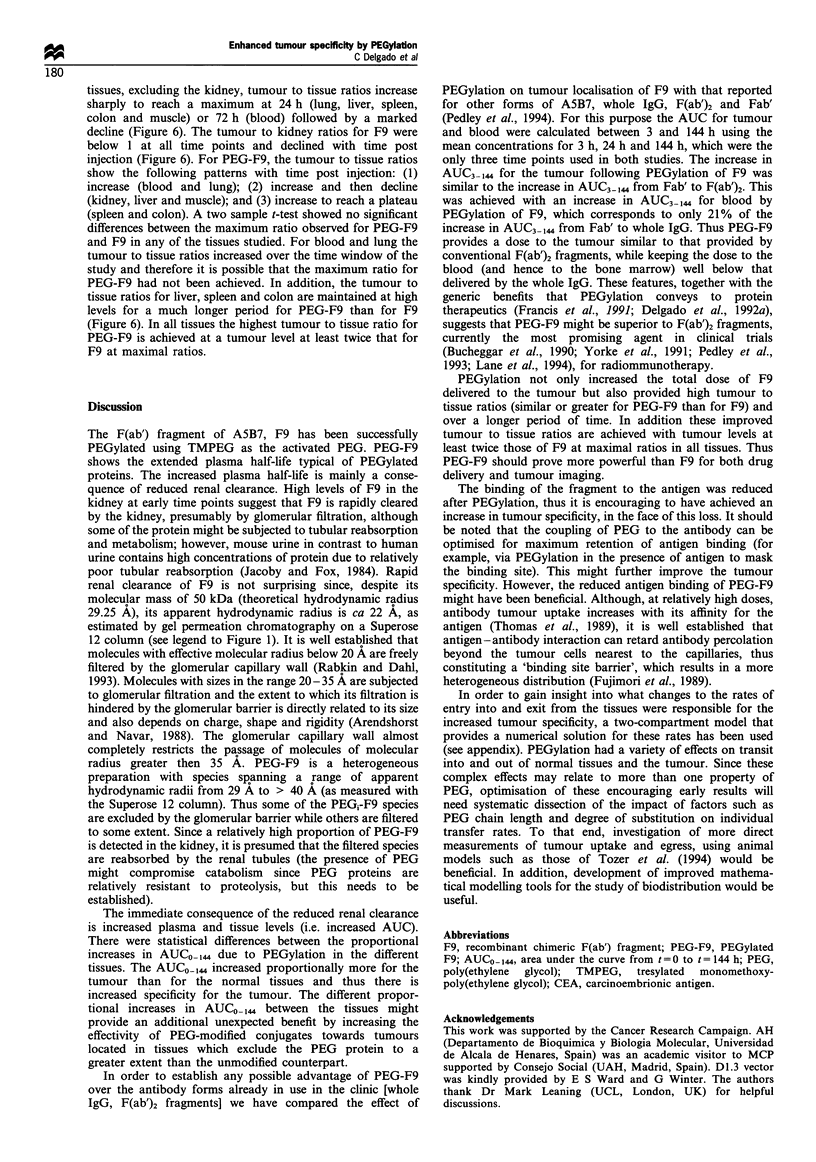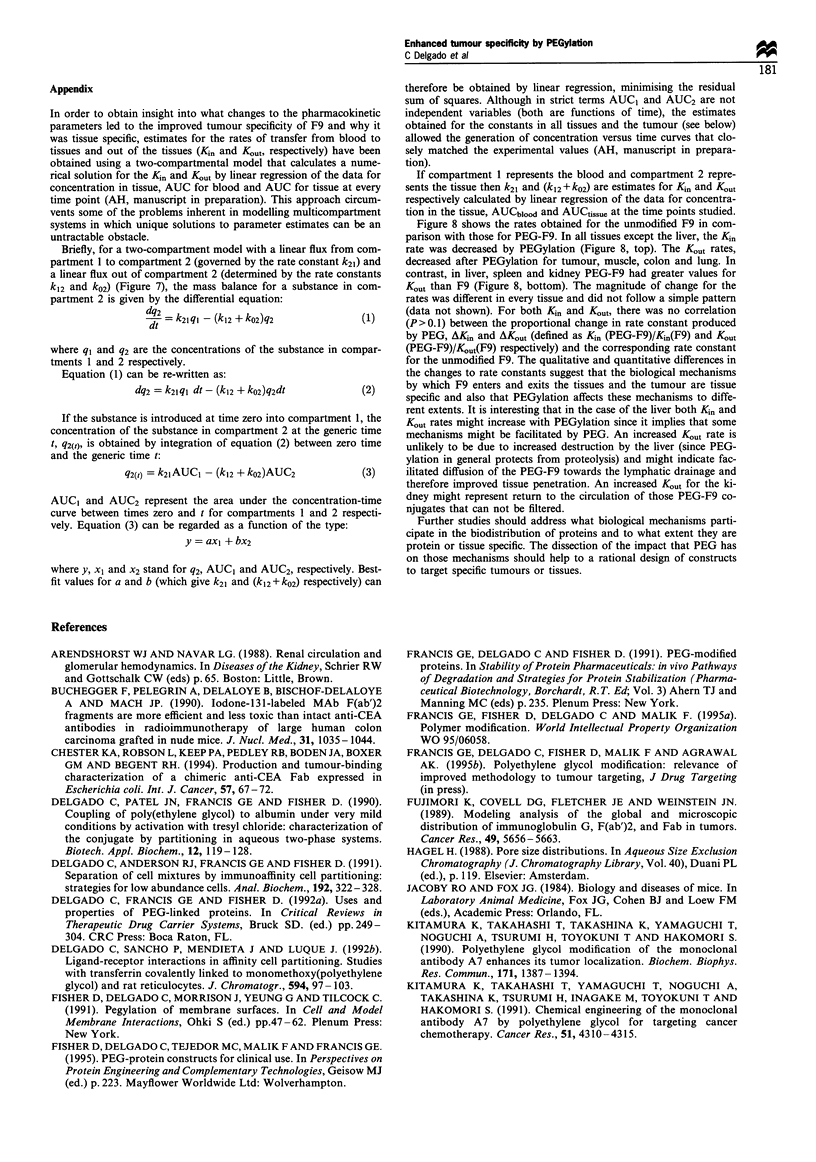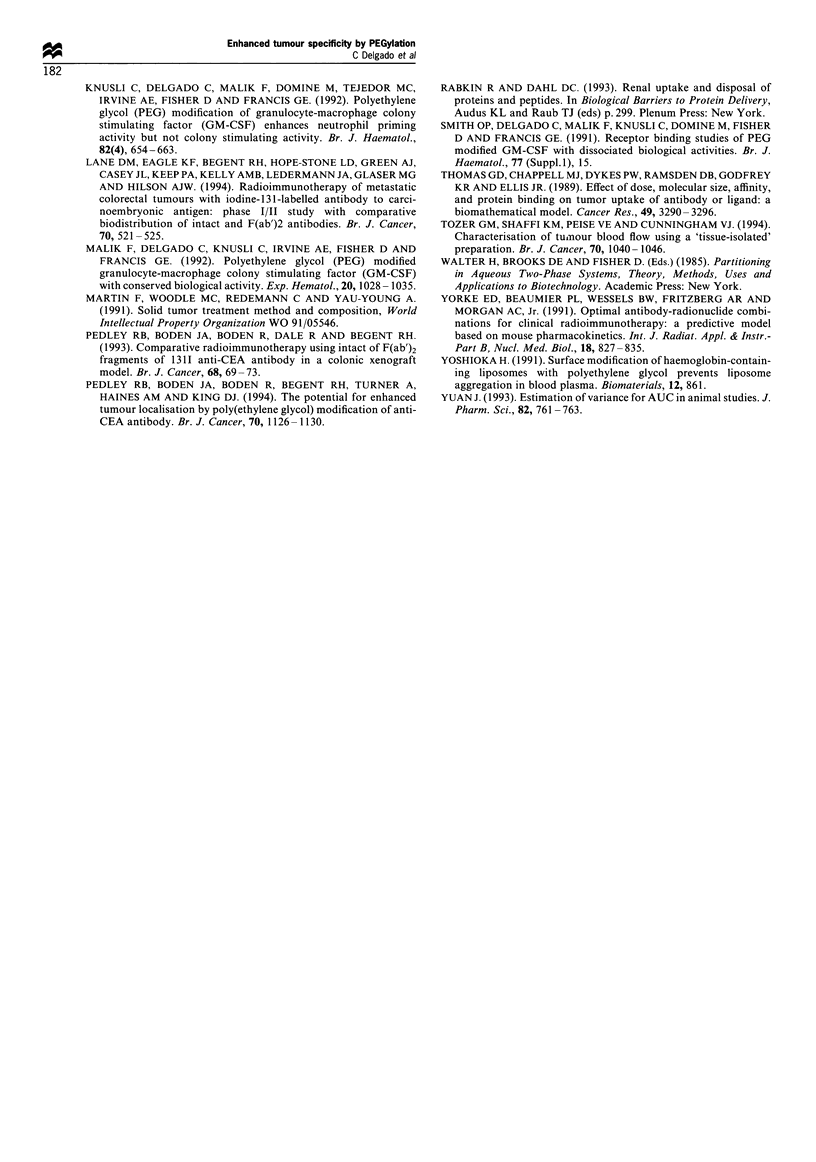# Enhanced tumour specificity of an anti-carcinoembrionic antigen Fab' fragment by poly(ethylene glycol) (PEG) modification.

**DOI:** 10.1038/bjc.1996.32

**Published:** 1996-01

**Authors:** C. Delgado, R. B. Pedley, A. Herraez, R. Boden, J. A. Boden, P. A. Keep, K. A. Chester, D. Fisher, R. H. Begent, G. E. Francis

**Affiliations:** Molecular Cell Pathology Laboratory, Royal Free Hospital School of Medicine, London, UK.

## Abstract

Polyethylene glycol (PEG) modification of a chimeric Fab' fragment (F9) of A5B7 (alpha-CEA), using an improved coupling method, increases its specificity for subcutaneous LS174T tumours. PEGylation increased the area under the concentration-time curve (AUC0-144) in all tissues but there were significant differences (variance ratio test, F = 27.95, P < 0.001) between the proportional increases in AUC0-144, with the tumour showing the greatest increase. The increase in AUCtumour from F9 to PEG-F9 was similar to the reported increase from Fab' to F(ab')2 while the increase in AUCblood by PEGylation of F9 was only 21% of the reported increase from Fab' to whole IgG. A two sample t-test showed no significant differences between maximal tumour/tissue ratios for PEG-F9 and F9 while the tumour/tissue ratios for PEG-F9 remained high over a longer period, with tumour levels at least double those for F9. PEG-F9 emerges as a new generation antibody with potential advantages for both radioimmunotherapy and tumour imaging. Since there was a reduction in antigen binding, optimisation of PEGylation might further improve tumour specificity. The latter resulted from complex effects on both the entry into and exit rates from tumour and normal tissues in a tissue-specific fashion.


British Journal of Cancer (1996) 73, 175–182
© 1996 Stockton Press All rights reserved 0007-0920/96 $12.00

### Enhanced tumour specificity of an anti-carcinoembrionic antigen Fab′ fragment by poly(ethylene glycol) (PEG) modification

C Delgado[1], RB Pedley[2], A Herraez[1], R Boden[2], JA Boden[2], PA Keep[2], KA Chester[2], D Fisher[1], RHJ Begent[2] and GE Francis[1]

[1]*Molecular Cell Pathology Laboratory and* [2]*CRC Targeting and Imaging Group (Clinical Oncology), Royal Free Hospital School of Medicine, Rowland Hill Street, London NW3, UK.*

**Summary** Polyethylene glycol (PEG) modification of a chimeric Fab′ fragment (F9) of A5B7 (α-CEA), using an improved coupling method, increases its specificity for subcutaneous LS174T tumours. PEGylation increased the area under the concentration–time curve ($AUC_{0-144}$) in all tissues but there were significant differences (variance ratio test, $F=27.95$, $P<0.001$) between the proportional increases in $AUC_{0-144}$, with the tumour showing the greatest increase. The increase in $AUC_{tumour}$ from F9 to PEG-F9 was similar to the reported increase from Fab′ to $F(ab')_2$ while the increase in $AUC_{blood}$ by PEGylation of F9 was only 21% of the reported increase from Fab′ to whole IgG. A two sample *t*-test showed no significant differences between maximal tumour/tissue ratios for PEG-F9 and F9 while the tumour/tissue ratios for PEG-F9 remained high over a longer period, with tumour levels at least double those for F9. PEG-F9 emerges as a new generation antibody with potential advantages for both radioimmunotherapy and tumour imaging. Since there was a reduction in antigen binding, optimisation of PEGylation might further improve tumour specificity. The latter resulted from complex effects on both the entry into and exit rates from tumour and normal tissues in a tissue-specific fashion.

**Keywords:** poly(ethylene glycol)-modification; tumour targeting; immunotherapy; pharmacokinetics

Covalent modification of proteins with poly(ethylene glycol)(PEG) has emerged as the method of choice to overcome the major problems associated with protein therapeutics (Francis *et al.*, 1991; Delgado *et al.*, 1992*a*). Increased plasma half-life, increased resistance to proteolysis and substantial reduction in antigenicity/immunogenicity have been found in almost all recombinant and native proteins after PEGylation. The success of this technology is indicated by the number of PEG proteins already in clinical trials (Francis *et al.*, 1991).

The benefits of PEG-modification might also extend beyond these recognised advantages, since PEG-coupled antibodies (Kitamura *et al.*, 1990, 1991) and PEG-coupled liposomes (Martin *et al.*, 1991) have recently been reported to have tumour-localising properties. The mechanisms leading to the improved localisation are, however, unclear. Whether the observed tumour-localising effect simply resulted from the longer circulation time or was related to some other consequence of PEGylation remains to be established. PEG changes the surface properties of the molecules and macromolecular structures to which it is attached (Fisher *et al.*, 1991); short range charge-based interactions are precluded (Fisher *et al.*, 1991) and there is a relationship between the extent to which this occurs and the length of the PEG (Yoshioka, 1991). In addition, PEG alters the solubility of molecules and this might influence the capacity of PEG constructs to cross the extracellular matrix and other structures, hence entry to and exit from different sites.

We have developed an improved method (Francis *et al.*, 1995*a*) for the attachment of PEG to proteins based on an earlier method that uses activation of the polymer with tresyl chloride (Delgado *et al.*, 1990). The tresylated PEG (TMPEG) has many advantages over PEGs activated by other methods (Delgado *et al.*, 1991; Francis *et al.*, 1991; Malik *et al.*, 1992). It links PEG to proteins using mild conditions for the coupling step, which results in a better conservation of biological activity; the activated polymer shows low toxicity, which allows addition of the whole reaction mixture to biological assay systems for a rapid test of the bioactivity of the PEG-protein conjugates (Fisher *et al.*, 1995; Francis *et al.*, 1995*b*). The PEG is attached directly to the molecule via a stable secondary amine linkage without any coupling moiety (portion of the activated PEG molecule remaining in the PEG-protein construct). The latter is particularly undesirable since coupling moieties can react linking further molecules, or be immunogenic/antigenic, are sometimes toxic and potentially cleaved enzymatically. The surface properties of the conjugate might also be affected by a coupling moiety (by altering the charge and/or the hydrophobicity/hydrophilicity balance). Our improved coupling method thus provides a unique tool with which to study the influence of PEGylation *per se* in tumour localisation.

We have PEG-modified a recombinant chimeric F(ab′) fragment (F9) with variable regions derived from A5B7 (a murine monoclonal antibody to human carcinoembryonic antigen) and human CH1 (gamma isotype) and C kappa constant regions using TMPEG. PEG-F9 has been used to investigate the effects of PEG-modification on tumour localisation using a colonic tumour xenograft, LS174T, grown in TO nude mice.

### Materials and methods

Chemicals were obtained from: MPEG (Mr 5000, Union Carbide, USA), PEG (Mr 6000, BDH, Poole, UK), dextran T-500 (Pharmacia, Sweden), tresyl chloride (Fluka, Switzerland). Phosphate-buffered saline (PBS) was from Gibco or Sigma (Poole, UK) and $^{125}I$ from Amersham (UK). Molecular weight markers were from Sigma. A kit for protein determination using the Coomassie brilliant blue assay was from Pierce (Rockford, IL, USA). All other reagents were Analar grade from BDH (Poole, UK).

### *Production of F9*

The protocol for the production of heavy chain (VH) and light chain (VL) cDNA of A5B7 and their subsequent cloning into the D1.3 Fab vector [containing the human C kappa and human CH1 (gamma isotype) constant regions] have been recently reported (Chester *et al.*, 1994). Briefly, VH was first

Correspondence: C Delgado
Received 24 April 1995; revised 17 August 1995; accepted 23 August 1995

cloned into *Pst*I and *Bst*EII sites in D1.3 and the resulting plasmid was used as the vector for insertion of VL into *Sac*I and *Xho*I sites. The constructs were transformed into *Escherichia coli* 'Sure' strain (Stratagene, La Jolla, CA, USA). The recombinant chimeric F(ab') fragment (F9) was isolated by affinity chromatography in a Sepharose 4B column linked to anti-human kappa and subsequent size exclusion chromatography in Sephacryl S-100. F9 gives positive staining for malignant glandular structures in human colorectal tumours consistent with staining for CEA (Chester *et al.*, 1994).

*Iodination of F9*

F9 was labelled with $^{125}$I by the chloramine T method to a sp. act. of ca 40 MBq $mg^{-1}$. Briefly, $^{125}$I was added to F9 in PBS followed by chloramine T. After 3 min incubation on ice L-tyrosine was added to stop incorporation to F9. The $^{125}$I-labelled F9 was isolated from the reaction mixture by gel filtration in Sephadex G-25 [PD-10 column from Pharmacia, primed with 0.1% bovine serum albumin (BSA)]. This iodination procedure has been shown to spare the integrity of the fragment in terms of size and binding affinity to CEA (Chester *et al.*, 1994).

*PEGylation of F9*

Monomethoxypolyethylene glycol (MPEG) of 5000 Mr was activated with tresyl chloride using a modified version (Francis *et al.*, 1995*a*) of our earlier method (Delgado *et al.*, 1990). MPEG is selected as the parent polymer to provide only one active end in each molecule, thus avoiding the formation of cross-linked products (although the polymer attached to F9 is MPEG, the conjugates are referred to as PEG-F9). This coupling method provides a direct linkage between the polymer and the protein via a stable secondary amine. The activated polymer, TMPEG, was incubated with $^{125}$I-labelled F9 (100 $\mu g\ ml^{-1}$) in PBS at room temperature for 2 h using a rotary mixer. The excess TMPEG was then inactivated by further 2 h incubation with a large excess of glycine. The preparation was kept at 4°C before use. Pharmacokinetic studies were performed with preparations stored for less than 12 h. The TMPEG-treated sample was always compared with $^{125}$I-labelled F9 subjected to the same procedures but substituting PBS for the polymer.

*Gel permeation chromatography*

Reaction mixtures and sham-treated controls were chromatographed on a Pharmacia fast protein liquid chromatography (FPLC) system fitted with a Superose-12 HR 10/30 column previously equilibrated with PBS. Since high concentrations of TMPEG in the reaction mixtures interfere with the resolution of the column (Malik *et al.*, 1992), all samples were diluted 1:50 in PBS before loading. Diluted samples (200 $\mu$l) were eluted at a flow rate of 0.3 ml per minute with sterile PBS; 0.25 ml fractions were collected. Protein concentration in the fractions was established by the levels of $^{125}$I quantified in a gamma counter. Molecular weight markers at concentrations of 3 mg $ml^{-1}$ were run similarly and the fractions analysed for protein content using a Coomassie Brilliant blue assay. Universal calibration of the column (i.e. hydrodynamic radius *vs* elution volume) using $\beta$-amylase (200 kDa), alcohol dehydrogenase (yeast, 150 kDa), BSA (66 kDa), carbonic anhydrase (29 kDa), cytochrome *c* (12.4 kDa) and aprotinin (6.5 kDa) was used to estimate the apparent hydrodynamic radius of F9 before and after PEGylation. To convert the mol. wt. of the protein markers to hydrodynamic radius, the relationship given by Hagel (1988) was used.

*Partition coefficient in PEG/dextran two-phase systems*

The two-phase system of 5% (w/w) PEG-6000, 5% (w/w) dextran T-500, 0.15 M sodium chloride and 0.01 M sodium phosphate [non-charge sensitive two-phase system, (Walter *et al.*, 1985)] was prepared in advance from stock solutions of 20% (w/w) dextran T-500, 40% (w/w) PEG-6000, 0.5 M sodium phosphate pH 6.8 and 0.6 M sodium chloride. After mixing, the system was allowed to settle into the top PEG-rich phase and the bottom dextran-rich phase at room temperature. The top and bottom phases were stored in separated bottles at 4°C until required. Before use the stored phases were allowed to equilibrate to room temperature and the biphasic system reconstituted by mixing top and bottom phases at a 1:1 volume ratio. Aliquots (10–50 $\mu$l) of reaction mixtures or sham-treated control were incorporated and the system mixed by 30–40 gentle inversions. After separation of the phases, aliquots from top and bottom phases were analysed for $^{125}$I levels. The partition coefficient ($K$) is calculated as the ratio between $^{125}$I levels in top and bottom phases.

In vivo *studies*

*Xenografts* All mice used were 2–3 months old female (TO nude) bearing a human colon adenocarcinoma xenograft grown in the flank for 2–3 weeks. The body weight of the animals was 20–25 g and the average size of the tumours was 0.625 g (mode 0.5–0.6 g). The xenograft tumour model was developed by subcutaneous inoculation of LS174T cells and subsequent passaging was by continuous subcutaneous implantation from the original xenograft (Pedley *et al.*, 1993). The tumour is a moderately differentiated, CEA-producing, adenocarcinoma with small glandular acini and which secretes no measurable CEA into the circulation.

*Biodistribution studies* Sham-treated F9 and PEG-F9 were administered intravenously (*ca* 8.5 $\mu$g) and at the indicated time points three mice per condition were bled and killed and the following organs removed: liver, kidney, lung, spleen, colon, muscle and tumour. The weight of the wet organ was recorded and the $^{125}$I content measured in a gamma counter (LKB, Bromma, Sweden, Wallac 1282 Compugamma). Animals were given food and water *ad libitum*, the water containing 0.1% potassium iodide in order to block thyroid uptake. Levels of F9 and PEG-F9 are given as percent injected radioactivity per g of blood and per cent injected radioactivity per g of wet tissue.

*Calculation of the $AUC_{0-144}$* was done by the linear trapezoid method using the mean tissue concentrations ($y$) at each time point ($t$). The following expression described by Yuan (1993) was used:

$$AUC_{t_0-t_n} = 1/2(t_1 - t_0)y_0 + \sum_{i=2}^{n} 1/2(t_i - t_{i-2})y_{i-1} + 1/2(t_n - t_{n-1})y_n$$

This expression shows that the estimated $AUC_{t_0-t_n}$ is a linear combination of mean concentrations at each time point (Yuan, 1993) and therefore the variance (s.d.$^2$) of the $AUC_{t_0-t_n}$ estimate can be calculated from the estimated standard error (s.e.m.) of the mean tissue concentrations at each time point using the expression described by Yuan (1993):

$$S.D.^2(AUC_{t_0-t_n}) = (1/2(t_1 - t_0)s.e.m._0)^2 + \sum_{i=2}^{n}(1/2(t_i - t_{i-2})\, s.e.m._{i-1})^2 + (1/2(t_n - t_{n-1})s.e.m._n)^2$$

*Statistical analysis*

The increments in $AUC_{0-144}$ promoted by PEGylation were compared using the $F$-test for the variance ratio. The maximal tumour to tissue ratios for F9 and PEG-F9 in each organ were compared using a two (unpaired) sample $t$-test.

*Binding studies*

Flat-bottomed microtitre plates receiving CEA in PBS (0.2 $\mu$g per well) were incubated for 1 h at room temperature. Wells were then washed with PBS and quenched with 3% BSA in PBS (150 μl per well) for *ca* 2 h at room temperature. Wells were washed twice with PBS before receiving the sample containing F9 or PEG-F9. Incubation was carried out overnight at room temperature and after washing three times with PBS containing 0.05% Tween 20 and four times with distilled water, the $^{125}I$ content in each well was measured in a gamma counter. Neither F9 nor PEG-F9 showed any binding to PBS-coated wells.

### Results

Incubation of F9 with TMPEG resulted in the formation of species with larger molecular sizes as shown by the appearance of faster eluting peaks in gel permeation chromatography (Figure 1). The proportion of larger molecular size species increased with the concentration of TMPEG in the reaction mixture, while the proportion of material eluting at the location of the unmodified F9 decreased (Figure 1). To confirm that the increase in molecular size was due to the formation of PEG-F9 conjugates, the reaction mixtures and sham-treated control (see methods) were analysed in a PEG/dextran two-phase system. The principle exploited here is that PEG-proteins have a higher affinity for the PEG-rich top phase than their unmodified counterparts (Delgado *et al.*, 1990, 1991). The partition coefficient (ratio between protein concentrations in top and bottom phases) of F9 in a 5% PEG-6000, 5% dextran T500, charge insensitive two-phase system was $1.14 \pm 0.08$ (mean ± s.e.m, $n = 5$). After incubation with TMPEG the partition coefficient increased to $4.01 \pm 0.42$ ($n = 3$) for a reaction mixture containing TMPEG at a molar excess of 75:1 (TMPEG: free amino groups) and $6.42 \pm 1.23$ ($n = 3$) when the molar excess of TMPEG was raised to 300:1. The increase in both molecular size and partition coefficient confirms the formation of PEG-F9 conjugates. The broadness of the FPLC profiles suggests that under all reaction conditions a mixture of $PEG_i$-F9 conjugates of a range of degrees of substitution (*i*) was produced. The increase in partition coefficient with the molar excess of TMPEG is consistent with an increase in the proportion of conjugates of higher degrees of substitution.

To test the influence of PEG modification on the tumour localisation of the antibody fragment, PEG-F9 conjugates were produced by incubation of F9 and TMPEG at a molar excess of TMPEG to $NH_2$ of 300:1 (Figure 1d). These reaction conditions were selected to minimise the contamination of the sample with unmodified F9 and to increase the proportion of species with molecular sizes exceeding the renal threshold.

The binding of PEG-F9 to the antigen was studied in comparison with that of F9 in order to establish how important this is for tumour localisation. Both untreated and control F9 (i.e. F9 exposed to 2 h incubation in PBS at room temperature) showed similar binding to the antigen which was greater than antigen binding for PEG-F9 (Figure 2). In this case, the reduced binding probably implies the presence of one or more lysines in, or near, the antigen-binding site. Reduction in receptor-binding affinity has previously been observed for proteins PEGylated by earlier TMPEG methods (Delgado *et al.*, 1992*b*; Smith *et al.*, 1991). However, this previous method gave relatively good conservation of

**Figure 2** Binding of untreated F9 (□), sham F9 (○) and PEG-F9 (●) to CEA in microtitre plates.

**Figure 1** Gel permeation chromatography of native F9 (**a**) and F9 incubated in PBS at a constant concentration of 100 μg ml$^{-1}$ with increasing concentrations of TMPEG to give TMPEG/$NH_2$ molar ratios of: 37.5:1 (**b**), 75:1 (**c**) and 300:1 (**d**). The Superose 12 column fitted to a FPLC system was loaded with 200 μl of 1:50 diluted reaction mixture or sham-treated control and eluted with PBS at a constant flow rate of 0.3 ml min$^{-1}$. Fractions of 0.25 ml were collected. The hydrodynamic radii calculated from the apparent mol. wts. obtained using globular proteins as standards as reported by Hagel (1988) was 22 Å for F9 and ranged from 29 Å to greater than 40 Å for PEG-F9 conjugates.

bioactivity (Knusli *et al.*, 1992; Malik *et al.*, 1992), now further improved by the modified method (Francis *et al.*, 1995*a*).

Figure 3 shows the blood clearance for F9 and PEG-F9 in TO nude mice bearing LS174T tumours. F9 was rapidly removed from the circulation and PEG-F9 showed the typical enhanced life in the circulation of PEG-modified proteins.

Figure 4 compares the concentrations of F9 and PEG-F9 in LS174T tumours subcutaneously implanted in the flank. F9 and PEG-F9 profiles in the tumour tissue run almost in parallel, albeit with higher levels for PEG-F9 than for F9 at all time points (Figure 4, solid lines). The $AUC_{0-144}$ estimates (per cent injected $^{125}I\ h\ g^{-1}$) were 82.87±15.95 for F9 and 472.17±80.53 for PEG-F9 (i.e. almost 6-fold increase in tumour 'dose'). Levels of F9 per g of tumour equalled levels of F9 per g of blood by 3 h post injection and by 10 h post injection tumour levels already exceeded blood levels (Figure 4). For PEG-F9 tumour levels only exceeded blood levels at a later time point, between 10 and 24 h post injection (Figure 4). It should be noted that for F9, tumour/blood ratios rise and then fall, whereas for PEG-F9 tumour/blood ratios increase progressively from 24 to 144 h (see below).

Figure 5 shows the biodistribution of F9 and PEG-F9 to kidney, liver, spleen, lung, colon and muscle. In all tissues the levels of PEG-F9 were above the levels of F9 at virtually all time points although the proportional increase varied from tissue to tissue and also, in contrast to the tumour, with time post injection (i.e. biodistribution profiles for F9 and PEG-F9 do not run in parallel) (Figure 5, solid lines). The time beyond which tissue levels exceed blood levels was tissue

**Figure 3** Disappearance of F9 (○) and PEG-F9 (●) from the blood following intravenous administration to TO nude mice bearing LS174T tumours subcutaneously implanted in the flank. Data shows mean±s.e.m., $n=3$ (error bars for some points were obscured by the symbol). The lines fitted are spline curves.

**Figure 4** Distribution of F9 (○) and PEG-F9 (●) to LS174T tumours subcutaneously implanted in the flank of TO nude mice after intravenous administration. The dashed and dotted lines show blood levels (mean values shown in Figure 2) for F9 and PEG-F9 respectively. Data shows mean±s.e.m., $n=3$ (error bars for some points were obscured by the symbol). The lines fitted are spline curves.

**Figure 5** Distribution of F9 (○) and PEG-F9 (●) to kidney, liver, spleen, lung, colon and muscle following intravenous administration to TO nude mice bearing LS174T tumours subcutaneously implanted in the flank. The dashed and dotted lines show blood levels (mean values shown in Figure 2) for F9 and PEG-F9 respectively. Data shows mean±s.e.m., $n=3$ (error bars for some points are obscured by the symbol). The lines fitted are spline curves.

specific and for all tissues longer for PEG-F9 than for F9 (Figure 5) thus PEG modification altered the pharmacokinetics of F9 in a tissue-specific fashion (a mathematical analysis of these changes is given in the appendix).

The practical consequences of all the changes in the pharmacokinetics have been summarised by comparison of: (a) the $AUC_{0-144}$ for F9 and PEG-F9 in each normal tissue and the tumour (Table I), (b) the proportional increase in $AUC_{0-144}$ for tumour and normal tissues by PEGylation (Table I) and (c) the tumour/blood and tumour/tissue ratios at each time point (Figure 6).

| | $AUC_{F9}$ (mean ± s.d.) | $AUC_{PEG\text{-}F9}$ (mean ± s.d.) | $AUC_{PEG\text{-}F9}/AUC_{F9}$ (mean ± s.d.) |
|---|---|---|---|
| Tumour | 82.67 ± 15.95 | 472.17 ± 80.53 | 5.71 ± 1.47 |
| Liver | 31.88 ± 5.13 | 136.84 ± 34.39 | 4.29 ± 1.28 |
| Kidney | 204.79 ± 40.19 | 394.16 ± 105.03 | 1.92 ± 0.64 |
| Spleen | 34.16 ± 5.79 | 118.09 ± 30.61 | 3.45 ± 1.07 |
| Lung | 33.91 ± 7.36 | 156.12 ± 41.59 | 4.60 ± 1.58 |
| Colon | 18.89 ± 3.76 | 86.91 ± 30.33 | 4.60 ± 1.85 |
| Muscle | 8.64 ± 2.14 | 29.49 ± 8.49 | 3.41 ± 1.29 |

[a]Per cent injected radioactivity h $g^{-1}$ tissue

**Figure 6** Change in tumour/tissue ratios for F9 (○) and PEG-F9 (●) with time post injection. Data shows mean ± s.e.m., $n = 3$ (error bars for some points are obscured by the symbol).

The tumour is the tissue receiving the greatest dose of PEG-F9 followed by kidney (Table I). Liver, spleen, lung, colon and muscle received a much lower dose of PEG-F9, at least a third of the tumour dose (Table I). With unmodified F9 the kidney received the greatest dose and although tumour received the second highest dose, it was *ca* five times smaller than that received by the kidney (Table I). Liver, spleen and lung received similar doses of F9 and colon and muscle received lower doses (Table I). PEGylation increased the dose delivered to all tissues (Table I) but the variance ratio test shows that there are significant ($F = 27.95$, $P < 0.001$) differences between the proportional increases in $AUC_{0-144}$ due to PEGylation, with tumour showing the highest increment. Thus PEG modification improves the specificity of F9 for the tumour. The increments in $AUC_{0-144}$ were unrelated to the dose of unmodified F9 received by each tissue (data not shown).

The tumour/tissue ratios were also analysed as a function of time post injection (Figure 6). For F9 in all the normal

**Figure 7** Schematic representation of the two-compartmental model. The substance is introduced in compartment 1 and allowed to enter compartment 2 by a linear flux governed by $k_{21}$. The exit from compartment 2 comprises return to compartment 1 with a rate constant $k_{12}$ and destruction with a rate constant $k_{02}$. The exit rate from compartment 1, other than only to compartment 2 (dashed arrow) is not required for the model.

**Figure 8** Rates of transfer from blood to tissue ($K_{in}$) (**a**) and out of tissue ($K_{out}$) (**b**) for F9 (□) and PEG-F9 (■).

tissues, excluding the kidney, tumour to tissue ratios increase sharply to reach a maximum at 24 h (lung, liver, spleen, colon and muscle) or 72 h (blood) followed by a marked decline (Figure 6). The tumour to kidney ratios for F9 were below 1 at all time points and declined with time post injection (Figure 6). For PEG-F9, the tumour to tissue ratios show the following patterns with time post injection: (1) increase (blood and lung); (2) increase and then decline (kidney, liver and muscle); and (3) increase to reach a plateau (spleen and colon). A two sample *t*-test showed no significant differences between the maximum ratio observed for PEG-F9 and F9 in any of the tissues studied. For blood and lung the tumour to tissue ratios increased over the time window of the study and therefore it is possible that the maximum ratio for PEG-F9 had not been achieved. In addition, the tumour to tissue ratios for liver, spleen and colon are maintained at high levels for a much longer period for PEG-F9 than for F9 (Figure 6). In all tissues the highest tumour to tissue ratio for PEG-F9 is achieved at a tumour level at least twice that for F9 at maximal ratios.

### Discussion

The F(ab') fragment of A5B7, F9 has been successfully PEGylated using TMPEG as the activated PEG. PEG-F9 shows the extended plasma half-life typical of PEGylated proteins. The increased plasma half-life is mainly a consequence of reduced renal clearance. High levels of F9 in the kidney at early time points suggest that F9 is rapidly cleared by the kidney, presumably by glomerular filtration, although some of the protein might be subjected to tubular reabsorption and metabolism; however, mouse urine in contrast to human urine contains high concentrations of protein due to relatively poor tubular reabsorption (Jacoby and Fox, 1984). Rapid renal clearance of F9 is not surprising since, despite its molecular mass of 50 kDa (theoretical hydrodynamic radius 29.25 Å), its apparent hydrodynamic radius is *ca* 22 Å, as estimated by gel permeation chromatography on a Superose 12 column (see legend to Figure 1). It is well established that molecules with effective molecular radius below 20 Å are freely filtered by the glomerular capillary wall (Rabkin and Dahl, 1993). Molecules with sizes in the range 20–35 Å are subjected to glomerular filtration and the extent to which its filtration is hindered by the glomerular barrier is directly related to its size and also depends on charge, shape and rigidity (Arendshorst and Navar, 1988). The glomerular capillary wall almost completely restricts the passage of molecules of molecular radius greater then 35 Å. PEG-F9 is a heterogeneous preparation with species spanning a range of apparent hydrodynamic radii from 29 Å to > 40 Å (as measured with the Superose 12 column). Thus some of the $PEG_i$-F9 species are excluded by the glomerular barrier while others are filtered to some extent. Since a relatively high proportion of PEG-F9 is detected in the kidney, it is presumed that the filtered species are reabsorbed by the renal tubules (the presence of PEG might compromise catabolism since PEG proteins are relatively resistant to proteolysis, but this needs to be established).

The immediate consequence of the reduced renal clearance is increased plasma and tissue levels (i.e. increased AUC). There were statistical differences between the proportional increases in $AUC_{0-144}$ due to PEGylation in the different tissues. The $AUC_{0-144}$ increased proportionally more for the tumour than for the normal tissues and thus there is increased specificity for the tumour. The different proportional increases in $AUC_{0-144}$ between the tissues might provide an additional unexpected benefit by increasing the effectivity of PEG-modified conjugates towards tumours located in tissues which exclude the PEG protein to a greater extent than the unmodified counterpart.

In order to establish any possible advantage of PEG-F9 over the antibody forms already in use in the clinic [whole IgG, $F(ab')_2$ fragments] we have compared the effect of PEGylation on tumour localisation of F9 with that reported for other forms of A5B7, whole IgG, $F(ab')_2$ and Fab' (Pedley *et al.*, 1994). For this purpose the AUC for tumour and blood were calculated between 3 and 144 h using the mean concentrations for 3 h, 24 h and 144 h, which were the only three time points used in both studies. The increase in $AUC_{3-144}$ for the tumour following PEGylation of F9 was similar to the increase in $AUC_{3-144}$ from Fab' to $F(ab')_2$. This was achieved with an increase in $AUC_{3-144}$ for blood by PEGylation of F9, which corresponds to only 21% of the increase in $AUC_{3-144}$ from Fab' to whole IgG. Thus PEG-F9 provides a dose to the tumour similar to that provided by conventional $F(ab')_2$ fragments, while keeping the dose to the blood (and hence to the bone marrow) well below that delivered by the whole IgG. These features, together with the generic benefits that PEGylation conveys to protein therapeutics (Francis *et al.*, *1991*; Delgado *et al.*, 1992*a*), suggests that PEG-F9 might be superior to $F(ab')_2$ fragments, currently the most promising agent in clinical trials (Bucheggar *et al.*, 1990; Yorke *et al.*, 1991; Pedley *et al.*, 1993; Lane *et al.*, 1994), for radiommunotherapy.

PEGylation not only increased the total dose of F9 delivered to the tumour but also provided high tumour to tissue ratios (similar or greater for PEG-F9 than for F9) and over a longer period of time. In addition these improved tumour to tissue ratios are achieved with tumour levels at least twice those of F9 at maximal ratios in all tissues. Thus PEG-F9 should prove more powerful than F9 for both drug delivery and tumour imaging.

The binding of the fragment to the antigen was reduced after PEGylation, thus it is encouraging to have achieved an increase in tumour specificity, in the face of this loss. It should be noted that the coupling of PEG to the antibody can be optimised for maximum retention of antigen binding (for example, via PEGylation in the presence of antigen to mask the binding site). This might further improve the tumour specificity. However, the reduced antigen binding of PEG-F9 might have been beneficial. Although, at relatively high doses, antibody tumour uptake increases with its affinity for the antigen (Thomas *et al.*, 1989), it is well established that antigen–antibody interaction can retard antibody percolation beyond the tumour cells nearest to the capillaries, thus constituting a 'binding site barrier', which results in a more heterogeneous distribution (Fujimori *et al.*, 1989).

In order to gain insight into what changes to the rates of entry into and exit from the tissues were responsible for the increased tumour specificity, a two-compartment model that provides a numerical solution for these rates has been used (see appendix). PEGylation had a variety of effects on transit into and out of normal tissues and the tumour. Since these complex effects may relate to more than one property of PEG, optimisation of these encouraging early results will need systematic dissection of the impact of factors such as PEG chain length and degree of substitution on individual transfer rates. To that end, investigation of more direct measurements of tumour uptake and egress, using animal models such as those of Tozer *et al.* (1994) would be beneficial. In addition, development of improved mathematical modelling tools for the study of biodistribution would be useful.

### Abbreviations

F9, recombinant chimeric F(ab') fragment; PEG-F9, PEGylated F9; $AUC_{0-144}$, area under the curve from $t=0$ to $t=144$ h; PEG, poly(ethylene glycol); TMPEG, tresylated monomethoxy-poly(ethylene glycol); CEA, carcinoembrionic antigen.

### Acknowledgements

This work was supported by the Cancer Research Campaign. AH (Departamento de Bioquimica y Biologia Molecular, Universidad de Alcala de Henares, Spain) was an academic visitor to MCP supported by Consejo Social (UAH, Madrid, Spain). D1.3 vector was kindly provided by E S Ward and G Winter. The authors thank Dr Mark Leaning (UCL, London, UK) for helpful discussions.

### Appendix

In order to obtain insight into what changes to the pharmacokinetic parameters led to the improved tumour specificity of F9 and why it was tissue specific, estimates for the rates of transfer from blood to tissues and out of the tissues ($K_{in}$ and $K_{out}$, respectively) have been obtained using a two-compartmental model that calculates a numerical solution for the $K_{in}$ and $K_{out}$ by linear regression of the data for concentration in tissue, AUC for blood and AUC for tissue at every time point (AH, manuscript in preparation). This approach circumvents some of the problems inherent in modelling multicompartment systems in which unique solutions to parameter estimates can be an untractable obstacle.

Briefly, for a two-compartment model with a linear flux from compartment 1 to compartment 2 (governed by the rate constant $k_{21}$) and a linear flux out of compartment 2 (determined by the rate constants $k_{12}$ and $k_{02}$) (Figure 7), the mass balance for a substance in compartment 2 is given by the differential equation:

$$\frac{dq_2}{dt} = k_{21}q_1 - (k_{12} + k_{02})q_2 \qquad (1)$$

where $q_1$ and $q_2$ are the concentrations of the substance in compartments 1 and 2 respectively.

Equation (1) can be re-written as:

$$dq_2 = k_{21}q_1\ dt - (k_{12} + k_{02})q_2dt \qquad (2)$$

If the substance is introduced at time zero into compartment 1, the concentration of the substance in compartment 2 at the generic time $t$, $q_{2(t)}$, is obtained by integration of equation (2) between zero time and the generic time $t$:

$$q_{2(t)} = k_{21}\text{AUC}_1 - (k_{12} + k_{02})\text{AUC}_2 \qquad (3)$$

$\text{AUC}_1$ and $\text{AUC}_2$ represent the area under the concentration-time curve between times zero and $t$ for compartments 1 and 2 respectively. Equation (3) can be regarded as a function of the type:

$$y = ax_1 + bx_2$$

where $y$, $x_1$ and $x_2$ stand for $q_2$, $\text{AUC}_1$ and $\text{AUC}_2$, respectively. Best-fit values for $a$ and $b$ (which give $k_{21}$ and $(k_{12}+k_{02})$ respectively) can therefore be obtained by linear regression, minimising the residual sum of squares. Although in strict terms $\text{AUC}_1$ and $\text{AUC}_2$ are not independent variables (both are functions of time), the estimates obtained for the constants in all tissues and the tumour (see below) allowed the generation of concentration versus time curves that closely matched the experimental values (AH, manuscript in preparation).

If compartment 1 represents the blood and compartment 2 represents the tissue then $k_{21}$ and $(k_{12}+k_{02})$ are estimates for $K_{in}$ and $K_{out}$ respectively calculated by linear regression of the data for concentration in the tissue, $\text{AUC}_{\text{blood}}$ and $\text{AUC}_{\text{tissue}}$ at the time points studied.

Figure 8 shows the rates obtained for the unmodified F9 in comparison with those for PEG-F9. In all tissues except the liver, the $K_{in}$ rate was decreased by PEGylation (Figure 8, top). The $K_{out}$ rates, decreased after PEGylation for tumour, muscle, colon and lung. In contrast, in liver, spleen and kidney PEG-F9 had greater values for $K_{out}$ than F9 (Figure 8, bottom). The magnitude of change for the rates was different in every tissue and did not follow a simple pattern (data not shown). For both $K_{in}$ and $K_{out}$, there was no correlation ($P>0.1$) between the proportional change in rate constant produced by PEG, $\Delta K_{in}$ and $\Delta K_{out}$ (defined as $K_{in}$ (PEG-F9)/$K_{in}$(F9) and $K_{out}$ (PEG-F9)/$K_{out}$(F9) respectively) and the corresponding rate constant for the unmodified F9. The qualitative and quantitative differences in the changes to rate constants suggest that the biological mechanisms by which F9 enters and exits the tissues and the tumour are tissue specific and also that PEGylation affects these mechanisms to different extents. It is interesting that in the case of the liver both $K_{in}$ and $K_{out}$ rates might increase with PEGylation since it implies that some mechanisms might be facilitated by PEG. An increased $K_{out}$ rate is unlikely to be due to increased destruction by the liver (since PEGylation in general protects from proteolysis) and might indicate facilitated diffusion of the PEG-F9 towards the lymphatic drainage and therefore improved tissue penetration. An increased $K_{out}$ for the kidney might represent return to the circulation of those PEG-F9 conjugates that can not be filtered.

Further studies should address what biological mechanisms participate in the biodistribution of proteins and to what extent they are protein or tissue specific. The dissection of the impact that PEG has on those mechanisms should help to a rational design of constructs to target specific tumours or tissues.

### References

ARENDSHORST WJ AND NAVAR LG. (1988). Renal circulation and glomerular hemodynamics. In *Diseases of the Kidney*, Schrier RW and Gottschalk CW (eds) p. 65. Boston: Little, Brown.

BUCHEGGER F, PELEGRIN A, DELALOYE B, BISCHOF-DELALOYE A AND MACH JP. (1990). Iodone-131-labeled MAb F(ab')2 fragments are more efficient and less toxic than intact anti-CEA antibodies in radioimmunotherapy of large human colon carcinoma grafted in nude mice. *J. Nucl. Med.*, **31**, 1035–1044.

CHESTER KA, ROBSON L, KEEP PA, PEDLEY RB, BODEN JA, BOXER GM AND BEGENT RH. (1994). Production and tumour-binding characterization of a chimeric anti-CEA Fab expressed in *Escherichia coli. Int. J. Cancer*, **57**, 67–72.

DELGADO C, PATEL JN, FRANCIS GE AND FISHER D. (1990). Coupling of poly(ethylene glycol) to albumin under very mild conditions by activation with tresyl chloride: characterization of the conjugate by partitioning in aqueous two-phase systems. *Biotech. Appl. Biochem.*, **12**, 119–128.

DELGADO C, ANDERSON RJ, FRANCIS GE AND FISHER D. (1991). Separation of cell mixtures by immunoaffinity cell partitioning: strategies for low abundance cells. *Anal. Biochem.*, **192**, 322–328.

DELGADO C, FRANCIS GE AND FISHER D. (1992*a*). Uses and properties of PEG-linked proteins. In *Critical Reviews in Therapeutic Drug Carrier Systems*, Bruck SD. (ed.) pp. 249–304. CRC Press: Boca Raton, FL.

DELGADO C, SANCHO P, MENDIETA J AND LUQUE J. (1992*b*). Ligand-receptor interactions in affinity cell partitioning. Studies with transferrin covalently linked to monomethoxy(polyethylene glycol) and rat reticulocytes. *J. Chromatogr.*, **594**, 97–103.

FISHER D, DELGADO C, MORRISON J, YEUNG G AND TILCOCK C. (1991). Pegylation of membrane surfaces. In *Cell and Model Membrane Interactions*, Ohki S (ed.) pp.47–62. Plenum Press: New York.

FISHER D, DELGADO C, TEJEDOR MC, MALIK F AND FRANCIS GE. (1995). PEG-protein constructs for clinical use. In *Perspectives on Protein Engineering and Complementary Technologies*, Geisow MJ (ed.) p. 223. Mayflower Worldwide Ltd: Wolverhampton.

FRANCIS GE, DELGADO C AND FISHER D. (1991). PEG-modified proteins. In *Stability of Protein Pharmaceuticals: in vivo Pathways of Degradation and Strategies for Protein Stabilization (Pharmaceutical Biotechnology, Borchardt, R.T. Ed*; Vol. 3) Ahern TJ and Manning MC (eds) p. 235. Plenum Press: New York.

FRANCIS GE, FISHER D, DELGADO C AND MALIK F. (1995*a*). Polymer modification. *World Intellectual Property Organization* WO 95/06058.

FRANCIS GE, DELGADO C, FISHER D, MALIK F AND AGRAWAL AK. (1995*b*). Polyethylene glycol modification: relevance of improved methodology to tumour targeting, *J Drug Targeting* (in press).

FUJIMORI K, COVELL DG, FLETCHER JE AND WEINSTEIN JN. (1989). Modeling analysis of the global and microscopic distribution of immunoglobulin G, F(ab')2, and Fab in tumors. *Cancer Res.*, **49**, 5656–5663.

HAGEL H. (1988). Pore size distributions. In *Aqueous Size Exclusion Chromatography (J. Chromatography Library*, Vol. 40), Duani PL (ed.), p. 119. Elsevier: Amsterdam.

JACOBY RO AND FOX JG. (1984). Biology and diseases of mice. In *Laboratory Animal Medicine*, Fox JG, Cohen BJ and Loew FM (eds.), Academic Press: Orlando, FL.

KITAMURA K, TAKAHASHI T, TAKASHINA K, YAMAGUCHI T, NOGUCHI A, TSURUMI H, TOYOKUNI T AND HAKOMORI S. (1990). Polyethylene glycol modification of the monoclonal antibody A7 enhances its tumor localization. *Biochem. Biophys. Res. Commun.*, **171**, 1387–1394.

KITAMURA K, TAKAHASHI T, YAMAGUCHI T, NOGUCHI A, TAKASHINA K, TSURUMI H, INAGAKE M, TOYOKUNI T AND HAKOMORI S. (1991). Chemical engineering of the monoclonal antibody A7 by polyethylene glycol for targeting cancer chemotherapy. *Cancer Res.*, **51**, 4310–4315.

KNUSLI C, DELGADO C, MALIK F, DOMINE M, TEJEDOR MC, IRVINE AE, FISHER D AND FRANCIS GE. (1992). Polyethylene glycol (PEG) modification of granulocyte-macrophage colony stimulating factor (GM-CSF) enhances neutrophil priming activity but not colony stimulating activity. *Br. J. Haematol.*, **82(4)**, 654-663.

LANE DM, EAGLE KF, BEGENT RH, HOPE-STONE LD, GREEN AJ, CASEY JL, KEEP PA, KELLY AMB, LEDERMANN JA, GLASER MG AND HILSON AJW. (1994). Radioimmunotherapy of metastatic colorectal tumours with iodine-131-labelled antibody to carcinoembryonic antigen: phase I/II study with comparative biodistribution of intact and F(ab')2 antibodies. *Br. J. Cancer*, **70**, 521-525.

MALIK F, DELGADO C, KNUSLI C, IRVINE AE, FISHER D AND FRANCIS GE. (1992). Polyethylene glycol (PEG) modified granulocyte-macrophage colony stimulating factor (GM-CSF) with conserved biological activity. *Exp. Hematol.*, **20**, 1028-1035.

MARTIN F, WOODLE MC, REDEMANN C AND YAU-YOUNG A. (1991). Solid tumor treatment method and composition, *World Intellectual Property Organization* WO 91/05546.

PEDLEY RB, BODEN JA, BODEN R, DALE R AND BEGENT RH. (1993). Comparative radioimmunotherapy using intact of $F(ab')_2$ fragments of 131I anti-CEA antibody in a colonic xenograft model. *Br. J. Cancer*, **68**, 69-73.

PEDLEY RB, BODEN JA, BODEN R, BEGENT RH, TURNER A, HAINES AM AND KING DJ. (1994). The potential for enhanced tumour localisation by poly(ethylene glycol) modification of anti-CEA antibody. *Br. J. Cancer*, **70**, 1126-1130.

RABKIN R AND DAHL DC. (1993). Renal uptake and disposal of proteins and peptides. In *Biological Barriers to Protein Delivery*, Audus KL and Raub TJ (eds) p. 299. Plenum Press: New York.

SMITH OP, DELGADO C, MALIK F, KNUSLI C, DOMINE M, FISHER D AND FRANCIS GE. (1991). Receptor binding studies of PEG modified GM-CSF with dissociated biological activities. *Br. J. Haematol.*, **77** (Suppl.1), 15.

THOMAS GD, CHAPPELL MJ, DYKES PW, RAMSDEN DB, GODFREY KR AND ELLIS JR. (1989). Effect of dose, molecular size, affinity, and protein binding on tumor uptake of antibody or ligand: a biomathematical model. *Cancer Res.*, **49**, 3290-3296.

TOZER GM, SHAFFI KM, PEISE VE AND CUNNINGHAM VJ. (1994). Characterisation of tumour blood flow using a 'tissue-isolated' preparation. *Br. J. Cancer*, **70**, 1040-1046.

WALTER H, BROOKS DE AND FISHER D. (Eds.) (1985). *Partitioning in Aqueous Two-Phase Systems, Theory, Methods, Uses and Applications to Biotechnology*. Academic Press: New York.

YORKE ED, BEAUMIER PL, WESSELS BW, FRITZBERG AR AND MORGAN AC, Jr. (1991). Optimal antibody-radionuclide combinations for clinical radioimmunotherapy: a predictive model based on mouse pharmacokinetics. *Int. J. Radiat. Appl. & Instr.-Part B, Nucl. Med. Biol.*, **18**, 827-835.

YOSHIOKA H. (1991). Surface modification of haemoglobin-containing liposomes with polyethylene glycol prevents liposome aggregation in blood plasma. *Biomaterials*, **12**, 861.

YUAN J. (1993). Estimation of variance for AUC in animal studies. *J. Pharm. Sci.*, **82**, 761-763.